# Web-based cognitive behavioral relapse prevention program with tailored feedback for people with methamphetamine and other drug use problems: protocol for a multicenter randomized controlled trial in Japan

**DOI:** 10.1186/s12888-016-0793-x

**Published:** 2016-04-04

**Authors:** Ayumi Takano, Yuki Miyamoto, Norito Kawakami, Toshihiko Matsumoto, Tomohiro Shinozaki, Takashi Sugimoto

**Affiliations:** Department of Psychiatric Nursing, Graduate School of Medicine, The University of Tokyo, 7-3-1 Hongo, Bunkyo-ku, Tokyo, 113-0033 Japan; Department of Mental Health, Graduate School of Medicine, The University of Tokyo, 7-3-1 Hongo, Bunkyo-ku, Tokyo, 113-0033 Japan; Department of Drug Dependence Research, National Institute of Mental Health, National Center of Neurology and Psychiatry, 4-1-1 Ogawa-Higashi, Kodaira, Tokyo, 187-8553 Japan; Department of Biostatistics, Graduate School of Medicine, The University of Tokyo, 7-3-1 Hongo, Bunkyo-ku, Tokyo, 113-0033 Japan; Department of Health Technology Assessment and Public Policy, Graduate School of Public Policy, The University of Tokyo, 7-3-1 Hongo, Bunkyo-ku, Tokyo, 113-0033 Japan

**Keywords:** Web-based, Internet, Drug dependence, Methamphetamine, Relapse prevention, Cognitive behavioral therapy, Motivational interviewing, Self-monitoring

## Abstract

**Background:**

Despite the effectiveness of psychosocial programs for recovery from drug use problems, there have been challenges in implementation of treatment. Internet-based and computerized approaches have been known to be effective in treatment dissemination. The study purpose is to assess the effects of a web-based psychosocial relapse prevention program with a multicenter randomized controlled trial.

**Methods:**

Recruitment began in January 2015 for outpatient participants diagnosed with drug abuse or dependence who have used a primary abused drug in the past year at psychiatric hospitals and a clinic. Participants are randomized either to a web-based relapse prevention program or a self-monitoring group. The intervention is a web-based relapse prevention program named “e-SMARPP” that consists of six relapse prevention program modules with tailored feedback from health care professionals and 8 weeks of self-monitoring. The content is adapted from a face-to-face relapse prevention program which is based on cognitive behavioral therapy and motivational enhancement. The primary outcomes are relapse risk assessed by the *Stimulant Relapse Risk Scale* (baseline, 2-, 5- and 8-month) and the longest duration of consecutive abstinent days from primary abused drug during the intervention. Secondary outcomes will include motivation to change, self-efficacy for drug use and craving, abstinent days in the past 28 or 56 days, quality of life, sense of coherence, cost of substance use, medical cost, retention of treatment and use of self-help group. Completion, usability and satisfaction of the program will be also assessed to explore feasibility. This study protocol was approved by the Ethics Committee of The University of Tokyo and each recruiting hospital and clinic.

**Discussion:**

To our knowledge, this study is the first clinical trial to assess the effects of a web-based therapeutic program for drug users in Japan. If successful, this program is a promising approach for drug user treatment in Japan, where the stigma toward drug users is strong. The results are also useful for researchers who want to know about programs for various substances, including methamphetamine.

**Trial Registration:**

This trial was registered with the University Hospital Medical Information Network clinical trial registry: UMIN000016075 (Date of registration: January 5, 2015).

## Background

### Drug use problems and implementation of treatment

Drug use problems have been a serious public health concern and illicit drug dependence is a global burden, accounting for 0.8 % of global all-cause disability adjusted life years in 2010 [1]. Drug dependence is highest in the age cohort of 20–29 years and adversely affects young adults [1, 2]. In Japan, drug use prevalence and drug related health problems have been much lower than that of other countries [3–5]. However, methamphetamine use disorders are the most prevalent in drug addiction treatment. Moreover, problems related to prescription drug use and overdose are serious, especially in Japanese female patients [6–8]. Moreover, outpatient treatment and community-based support for drug users have been very poor because of a zero-tolerance policy [6]. Accessible psychiatric treatment use is very limited, accounting for about 16 % of drug/alcohol use disorders [9]. Because pharmacotherapy for drug dependence (e.g., methadone, buprenorphine) has not been approved in Japan, psychosocial approaches are the most important treatment, especially in outpatient settings. However, there has been a gap between potential treatment needs and available treatment services, which is also apparent in other countries. Various reasons have been considered as barriers to treatment access: (1) limited availability (e.g., rigid session times, inconvenient locations, costs for drug users), (2) concerns about confidentiality and stigmatization and (3) economic and human-resource limitations for treatment providers [10–14]. Flexible and accessible treatments are necessary, especially in Japan as outpatient treatment for drug users is very limited and societal drug-use stigma is strong.

### Treatment using computer and Internet technologies

Therapeutic interventions using computer and Internet technologies have developed and adapted to various health problems, including substance use disorders, to address challenges in treatment implementation [15–17]. Many computer-assisted or web-based interventions for drug users which were developed based on psychosocial approaches demonstrated benefits for abstinence, treatment retention and cost effectiveness with small to moderate effect sizes [10, 11, 18–22]. Most of these interventions have been developed for cocaine or cannabis users in Western countries. There are few programs for amphetamine-type-stimulant users and for populations in Asia with different social backgrounds, even when Internet infrastructure and computers are generally available [23, 24]. In Japan, there have been various web and mobile applications to assist in personal health care, however, evidence-based therapeutic interventions for drug users remains undeveloped.

### Previous work: development of “e-SMARPP”

A new piloted web-based program named “e-SMARPP” for Japanese drug users was developed by the first author (AT) based on an existing face-to-face cognitive behavioral relapse prevention program [25], using Moodle (version 2.6.1) which is an open-source web application to build e-learning websites [26]. A referenced program was the Serigaya Methamphetamine Relapse Prevention Program (SMARPP), which was developed based on the Matrix Model by one of the authors (TM) and was widely implemented in Japan [25]. The web-based e-SMARPP program consisted of three parts: a relapse prevention program series that included videos, with narration and subtitles, and assignments in the form of exercises and a diary; self-monitoring; and information about drug addiction services. e-SMARPP content is intended to be user-friendly with minimal text and limited use of difficult Kanji characters referencing specialized medical terminology. User guides in each section support use. The e-SMARPP website is designed to support any device, including personal computers, mobile phones and tablet computers with Internet access. The website is closed access and only drug users diagnosed with drug dependence by psychiatrists are provided a login account from an administrator. The access security is protected by an individual login/password and secure socket layer technology. The content of e-SMARPP does not depend on the type of drug and was developed with versatility to assist in handling common problems among drug users. This was because most face-to-face programs for drug users deal with problems of various drugs and it was not feasible to gather homogeneous patients in Japan. The usability and acceptance of e-SMARPP were reasonable among psychiatric outpatients and people who had recovered from drug dependence, but some improvements were suggested [25]. Details of the development process and findings of the pilot study were reported in a previous study [25]. In the revision process after the piloted usability test, content of the videos were simplified to focus on approaches to recovery rather than drug adverse effects. Self-monitoring was improved to be able to record detailed conditions about drug users if necessary (e.g., drug consumption, forms of used drugs, triggers of drug use). Functional defects including garbled characters on mobile phones were fixed.

### Objectives and hypotheses

The aim of this study is to examine the effects of a web-based cognitive behavioral relapse prevention program, revised as e-SMARPP, among Japanese psychiatric outpatients with methamphetamine and other drugs use problems with a multicenter randomized controlled trial (RCT) design. The primary hypothesis is that participants assigned to e-SMARPP will have reduced relapse risk and maintain a longer duration of consecutive abstinence (days) from a primary abused drug during the intervention compared to those who were randomized to self-monitoring only. The secondary hypothesis is that participants in the e-SMARPP group will report positive changes in motivation to change, self-efficacy for drug craving, abstinent days in the past 28 or 56 days (percentages and differences in change: pre-post), quality of life, sense of coherence, cost of alcohol and drug, retention of treatment, use of self-help group and psychiatric medical cost. In addition, completion, usability and satisfaction of the program will be assessed for utilization and feasibility test. This article describes the study protocol according to SPIRIT guidelines [27].

## Methods

### Trial design

**Fig. 1:**
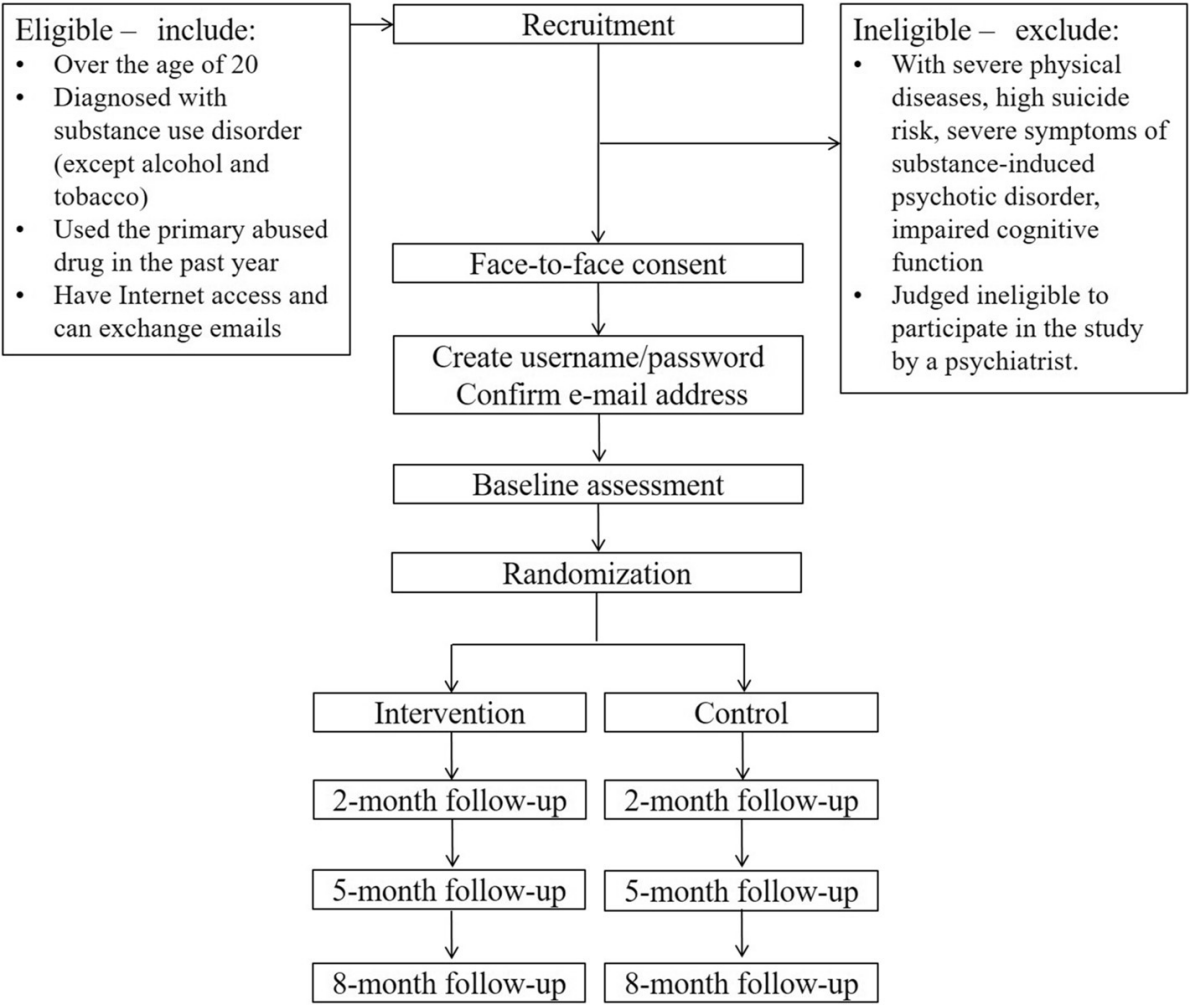
Study design and participant flow

Figure 1 shows the design of this study and participant flow. This study is a two-arm (allocation ratio is one to one), parallel-group, non-blinded and multicenter randomized controlled trial. Eligible participants are asked to complete the baseline assessment and are randomly allocated to either the intervention group or the control group. All participants in both groups will be provided a login/password with instructions about how to access the website and use e-SMARPP during the study. The participants in the intervention group can access the complete contents of e-SMARPP, while the control group can access a part of it: self-monitoring. Each individual access account is tied to either group, and as such participants can use e-SMARPP content included in their group only. Web-based follow-up assessments are conducted at 8, 20, 32 weeks after the baseline assessment.

### Participants and setting

The participants are recruited from five psychiatric hospitals and one clinic (National Center of Neurology and psychiatry, Saitama Psychiatric Medical Center, Kanagawa Psychiatric Center, Okayama Psychiatric Medical Center, Tokyo Metropolitan Matsuzawa Hospital and APARI clinic) that provides treatment for people with substance use disorder in Japan. The inclusion criteria are: (1) outpatients who were diagnosed with substance use disorder assessed by DSM-5 (psychoactive substances other than alcohol and tobacco), (2) those who used a primary abused drug in the past year and (3) those with access to the Internet via PC, smartphone or tablet computer and can exchange e-mail. The exclusion criteria are: (1) patients with severe physical diseases, (2) patients with high suicide risk, (3) patients with severe symptoms of substance-induced psychotic disorder, (4) patients with impaired cognitive function and (5) those who judged ineligible to participate in the study by a psychiatrist. We include various types of participants (form of drugs, previous and current receiving treatment for drug dependence, psychiatric comorbidity, pharmacotherapy and sexual orientation) because we will test adaptation of e-SMARPP to various drug users in a secondary analysis.

### Randomization and blinding

Staff of recruiting institutions will recruit outpatients who meet inclusion criteria. Eligible participants will be informed that they will be allocated to either of the two groups. After baseline assessment, they will be randomly assigned to either of the two groups using the method of permuted block, with random block size of four, and they will be informed their assigned group by the first author (AT). Randomization will be stratified by institution. The computer-generated allocation list was made by an independent researcher (YM) and concealed to other researchers and participants until the time of assignment. The enrollment is conducted by the first author (AT) and the intervention starts immediately. Researchers and staff who work for recruiting institutions will be blinded. In addition, an independent researcher (YM) who will not analyze data will download data from the e-SMARPP database and an independent research staff person will mask the group variable before analysis, then researchers (AT and T. Shinozaki) will analyze data that is blinded to the group variable.

### Interventions

#### Web-based relapse prevention program: e-SMARPP

The website of e-SMARPP is comprised of five modules: (1) cognitive behavioral relapse prevention sessions (watching videos, submitting exercises and a weekly dairy on the website), (2) self-monitoring, calendar that displays drug use status by color, (3) information, downloadable PDF and website links to drug addiction support services, (4) user guide, how to use the system, frequently asked questions and a contact form to researchers and (5) assessment, which are web-based questionnaires for baseline and three follow-up assessments.

**Fig. 2:**
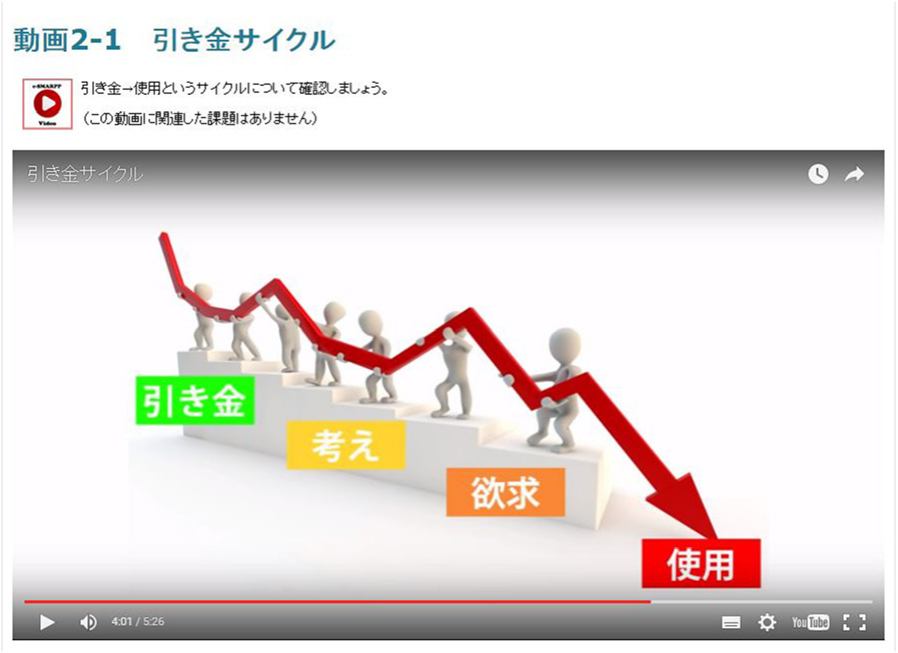
Screenshot of video

**Fig. 3:**
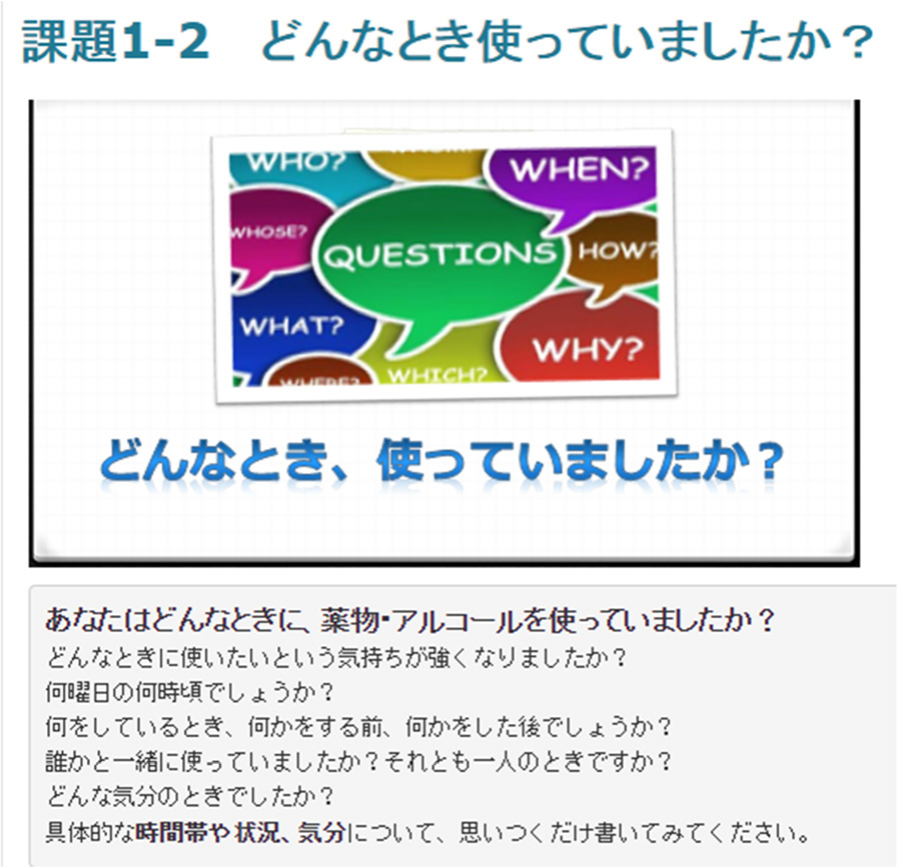
Screenshot of exercise

The main intervention modules are the relapse prevention program sessions and self-monitoring. Content for the videos and exercises of the relapse prevention program are taken from the SMARPP workbook and can be adapted to any type of drug. Each session has three videos, two exercises and a weekly diary activity (Table 1). Videos are made in a YouTube format and embedded in each session (See Fig. 2). Videos are online, but are unlisted videos and restricted to people who have the link to the video, so only participants in the intervention group and researchers can view them. Narration and subtitles help users understand the content. Exercises are related to the video content and users will be expected to complete these after watching the video. Users will write and submit their own answers through an Internet text form (See Fig. 3). In addition, users are expected to write down in the weekly diary their condition from the last week, current goals, and how they will plan to spend time over the next week. Writing in the diary is also done on the Internet through the system. After submitting the exercise and the weekly diary, users receive tailored feedback comments from qualified health care professionals (registered nurse and medical doctor) who are trained to support patients with substance use disorders (mainly AT). Feedback comments are based on motivational interviewing skills to enhance user motivation and to provide individual support.

**Table 1:** Content for relapse prevention session of e-SMARPP

1. What is drug dependence?	
Video	➢ Mental and physical consequences caused by drug use (11′ 02″)
	➢ Changes in the brain (11′ 39″)
	➢ How to stop a drug craving (7′ 43″)
Exercise	■ Think about your pros and cons for drug use and quitting drugs.
	■ Define your drug use situation: when, where, who, why, what and emotion.
2. Triggers of drug use	
Video	➢ Process of craving and drug use (5′ 27″)
	➢ Various internal and external triggers of drug craving (11′ 00″)
	➢ Anchors keeping you from drug use (5′ 01″)
Exercise	■ Define your internal and external triggers.
	■ Who and what are your anchors?
3. Recovery process; “Just for today”	
Video	➢ Process and stage of recovery (12′ 38″)
	➢ Safe lifestyle and signs of relapse (10′ 19″)
	➢ How to plan a safe daily life (9′ 27″)
Exercise	■ Think of your signs of relapse and barriers to recovery.
	■ Plan a safe daily life schedule without drugs.
4. Features of dependence symptoms	
Video	➢ Typical features of dependence (9′ 05″)
	➢ Typical thoughts and behaviors when people fall for drugs (12′ 32″)
	➢ Justification for relapse (9′ 21″)
Exercise	■ Think of your patterns of thinking and behavior during drug use
	■ Think of your possible justification for relapse
5. Supporters for recovery	
Video	➢ Typical internal triggers: “HALT” (hungry, angry, lonely and tired) (10′ 05″)
	➢ To trust and be honest to yourself and others (5′ 41″)
	➢ Support from peers and professionals (13′ 39″)
Exercise	■ Think of ways to handle internal triggers.
	■ Think of your supporters. Who? How to find?
6. No need to be strong, be smart and practiced	
Video	➢ Tips for recovery (6′ 04″)
	➢ Review of skills to handle triggers and relapse (12′ 21″)
	➢ To accept the way you are, messages from peers (4′ 32″)
Exercise	■ Think of crisis plans when you relapse into drug use.
	■ Think of your future when you recover from drug addiction.

Each session also includes a weekly diary activity

Parentheses indicate minutes and seconds of each video

**Fig. 4:**
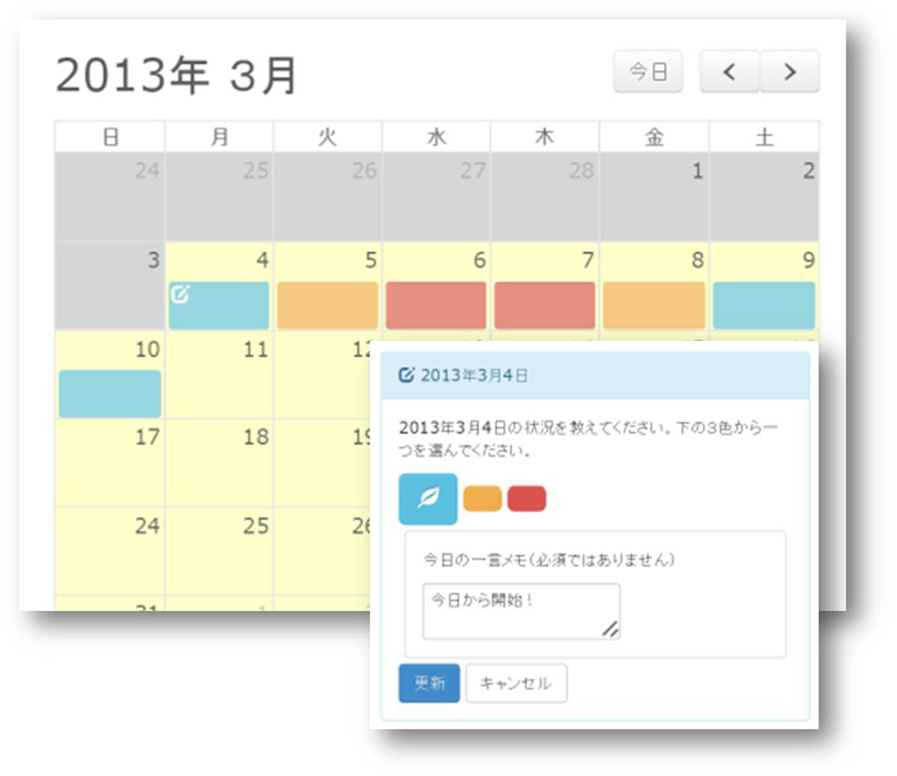
Screenshot of self-monitoring calendar

The self-monitoring is done in a calendar format, using a plug-in from Moodle, to provide a function similar to the self-monitoring process utilized in SMARPP. Users click on a date in the calendar and select one of three colors (red, yellow or blue), then that color subsequently displays on the date (See Fig. 4). The colors represent the user’s drug use: red reflecting abuse of the primary drug; yellow reflecting secondary abuse of other drugs and alcohol use; and blue indicating no drug and alcohol use. Instructions and a legend for colors are not displayed on the Web page to avoid concerns about confidentiality. Participants are provided an explanation about the colors and how to use the calendar at the time of study enrollment. This calendar is intended to record only presence or absence of drug use without quantity and frequency a day because primary abused drug will vary and the total quantity will not be able to be adequately compared. We prioritized a user-friendly system without many options for drug names and units. An optional memo function is provided for personal user use that records detailed conditions (drug form, quantity and frequency, triggers, etc.).

A self-monitoring calendar is used as a measure to assess drug use for a period of 28-days before the baseline and for follow-up assessments like the Timeline Followback (TLFB) method [28, 29]. Web-based versions of the TLFB methods have been developed and adapted to various substances with good reliability and validity [30–32] and have been used in some intervention studies [11, 19–21].

e-SMARPP is equipped with some automated functions for participants, including progress tracking and a notification e-mail when they receive feedback. In the notification e-mails, a related webpage link is shown and users can access the webpage directly. For example, users can view the feedback comment webpage directly after they click the link in the notification e-mail. Additionally, for researchers who registered on e-SMARPP, a notification e-mail will be sent when participants submit an exercise, diary and questionnaire.

#### Intervention group

Participants who are assigned to the intervention group are provided access to the complete contents of e-SMARPP, including six sessions for cognitive behavioral relapse prevention and web-based self-monitoring. They are expected to complete each session over a week in sequence order by each deadline (each Sunday). For an 8-week intervention period, they are expected to complete a total six sessions, but they have a 2-week grace period and are allowed to progress at their own pace. If they do not complete a session, the session will be carried over to the next week. Participants will be expected to record their daily situation of drug use on the web-based self-monitoring calendar by each deadline (each Sunday). If they do not go through an expected session and/or self-monitoring by each deadline, a researcher will send an e-mail reminder on the next day (Monday).

Participants continue to receive outpatient treatment as usual, including medication, face-to-face group or individual psychosocial treatment programs and counseling by psychologists and/or social workers. Provided treatment depends on individual condition. Even if participants stop receiving outpatient treatment or change their primary doctor and hospital, the web-based intervention will not be cancelled.

#### Control group

Participants who are assigned to the control group are provided access to a part of the contents of e-SMARPP, including the web-based self-monitoring and information content. Control group participants have no access to the cognitive behavioral relapse prevention sessions. Similar to the intervention group, they are expected to record their daily situation of drug use on the web-based self-monitoring calendar by each deadline (each Sunday). If they do not go through the self-monitoring by each deadline, a researcher will send an e-mail reminder on the next day (Monday). They will continue to receive outpatient treatment as usual similar to the intervention group. After the study period, cognitive behavioral relapse prevention sessions will be provided if requested.

### Measures

#### Data collection procedure

Table 2 shows an assessment schedule for this study. Data collection will be conducted through web-based self-reported questionnaires on the e-SMARPP website at baseline (T1) and follow-up assessments at 2 (T2), 5 (T3) and 8 (T4) month after the randomization. Participants will be informed about the follow-up assessments via e-mail and asked to complete the questionnaire within 1 week. After 1 week, an additional reminder e-mail will be sent to non-respondents. If a participant’s e-mail address changes and an e-mail is not received, a postcard will be sent as an extra reminder. Participants will receive a prepaid card for 1000 yen as a reward for baseline and each follow-up assessment that they complete.

**Table 2:** Assessment schedule of primary and secondary outcomes

Outcome		Measurement	Baseline (T1)	2-month follow-up (T2)	5-month follow-up (T3)	8-month follow-up (T4)
Primary outcome						
1	Relapse risk	SRRS	x	x	x	x
2	Longest consecutive duration of abstinence	Longest duration of consecutive abstinent days during the intervention #		x		
Secondary outcome						
1	Motivation to change	SOCRATES	x	x	x	x
2	Self-efficacy for handling drug use and craving	Self-efficacy Scale for Drug Dependence	x	x	x	x
3	Percentages of abstinent days	Abstinent days in the past 28 or 56 days #	x	x	x	x
4	Differences in change of abstinent days	Summed abstinent days in the past 28 days #	x	x	x	x
5	Health related quality of life	WHOQOL26	x	x	x	x
6	Sense of coherence	3-item sense of coherence scale	x	x	x	x
7	Cost of alcohol and drug	Self-reported cost of drugs or alcohol in the last month (yen)	x	x	x	x
8	Treatment retention	Yes or no	x	x	x	x
9	Self-help group use	Yes or no	x	x	x	x
10	Psychiatric medical cost	Self-reported medical use in the past six months	x			x
Feasibility and usability outcome						
1	Program completion	Number of completed weeks		x		
2	Satisfaction	CSQ-8		x		
3	Usability and usefulness	Original questionnaire		x		

*SRRS* Stimulant Relapse Risk Scale, *SOCRATES-8D* Stage of Change Readiness and Treatment Eagerness Scale-8 version for drug use, CSQ-8, 8-item Client Satisfaction Questionnaire

#: Self-reported drug use or abstinence assessed by web-based self-monitoring calendar or the Timeline Follow-back method

#### Primary outcome

The primary outcomes will be relapse risk, assessed using the Stimulant Relapse Risk Scale (SRRS) [33, 34], and the longest duration of consecutive abstinence (days). SRRS was developed to measure multidimensional relapse risk and consists of 35 items measured on a 3-point Likert scale. The SRRS has five subscales: anxiety and intention to use drug (AI), emotionality problems (EP), compulsivity for drug use (CD), positive expectancies and lack of control over drug (PL), and lack of negative expectancy for drug use (NE) [33]. All items ask about a drug-related situation in the past 1 week. Examples of the items are “I am anxious about reusing the drug” (AI), “I cannot control my feeling” (EP), “I want to obtain the drug even by working illegally” (CD), “If I use the drug, I would feel invigorated” (PL), and “If I use the drug, it would badly influence my job” (NE, inversed item). Higher average scores for total and subscale items indicate higher relapse risk. Its reliability and validity was confirmed among stimulant drug users in Japan [33].

We will add another primary outcome, the longest duration of consecutive abstinence (days), according to previous studies [19–21]. This is because the cut-off point of the SRRS has not been confirmed and interpretation of the score is difficult. Additionally, the SRRS was developed and is only used in Japan, and as such, a more objective outcome measure is needed. The longest duration of consecutive abstinence from the primary abused drug during intervention (56 days) will be counted, using the self-monitoring calendar and the TLFB method.

We hypothesize that both of the two primary outcomes will differ significantly between groups.

#### Secondary outcome

The secondary outcomes (Table 2) will be the following ten measures.

##### Motivation to change

Motivation to change will be measured with the Stage of Change Readiness and Treatment Eagerness Scale-8 version for Drug Use (SOCRATES-8D) [35, 36]. The SOCRATES-8D consists of 19 items assessed on a 5-point Likert scale and three subscales: Recognition, Ambivalence, and Taking Steps. Examples of the items are “I really want to make changes in my use of drugs” (Recognition), “Sometimes I wonder if I am an addict” (Ambivalence) and “I have already started making some changes in my use of drugs” (Taking Step). Higher scores indicate higher motivation to change. Positive correlations have been reported between high scores and the development of readiness for treatment [37] and engagement in treatment [38]. Reliability and validity of the Japanese version of the SOCRATES-8D has been confirmed [35, 39]. However, a different factor structure (two-factor structure) was observed in the previous study [35], so we will use only the total score in this study.

##### Self-efficacy

Confidence (i.e., self-efficacy) in handling drug use and craving is measured with the Self-efficacy Scale for Drug Dependence (SSDD) [40]. The SSDD has two domains: general self-efficacy (GE) and self-efficacy in specific situations (SS). The GE domain consists of five items assessed on a 5-point Likert scale from 1 (not confident) to 5 (confident). Examples of GE items include, “I can seek help when I have a problem”. The SS domain consists of 11 items assessed on a 7-point Likert scale from 1 (not at all confident) to 7 (absolutely confident). Examples of an SS item includes, “I can handle a drug craving when I am depressed and anxious”. Higher GE and SS scores mean more confidence in handling a drug craving.

##### Abstinence

Abstinence from the primary abused drug will be measured using the following three methods. First, percentages of abstinent days from the primary abused drug in the past 28 or 56 days at each assessment point will be compared. Next, differences in change of abstinent days from the primary abused drug in the past 28 days between baseline (T1) and follow-up (T2, T3 and T4) will be assessed. Finally, the longest consecutive abstinent days during the intervention period will be compared. Abstinent days will be summed using self-monitoring and the TLFB method.

##### Quality of life

Health related quality of life measured with WHO/QOL-26 [41], which consist of 26 items measured on a 5-point Likert scale. There are two items which asks about an individual’s overall perception of quality of life (QOL) and their health. The remaining 24 items are divided into four domains: physical domain, psychological domain, social relationships and environment. All items ask about respondents’ life in the last 4 weeks. Higher scores indicate higher QOL.

##### Sense of coherence

Sense of coherence (SOC) is considered to be an individual’s personality as a fundamental source of coping in stressful events [42]. The SOC of people with substance use disorder has been considered lower than that of healthy people [43]. Among people with mental health problems and substance use disorders, previous studies have revealed that high SOC is associated with a better ability to cope with stressful life situations and improved life satisfaction [43, 44] and high SOC is one of the predictors of treatment success: treatment retention and drug abstinence [45]. We will use the University of Tokyo Health Sociology version of the SOC3 scale (SOC-3-UTHS) [46, 47], which consists of three items measured on a 7-point Likert scale. A higher score indicates a higher SOC.

##### Treatment retention and alternative treatment use

We also will assess participants’ retention of outpatient treatment and use of self-help group such as narcotics anonymous.

##### Cost of substance use and treatment use

Total cost of alcohol and drug use in the past month will be asked separately. In Japan, methamphetamine is more expensive than other drugs (above 10,000 yen for use of methamphetamine several times). New psychoactive substances (NPS) are relatively cheap (about 2000 to 5000 yen for one package).

Cost of psychiatric treatment use in the past 6 months will also be assessed. We will ask about frequency and period of psychiatry hospitalization and outpatient treatment, frequency of use of emergency room, total cost of prescription drugs, frequency and period of specialized outpatient treatment for drug addiction, the participant’s income and amount of time required to receive one outpatient treatment including travel time. We will calculate total psychiatric medical cost using the annual report of medical costs by diseases and treatment [48].

#### Users’ feedback, feasibility, usability

In addition, completion, usability and satisfaction of the program will be assessed for utilization and a feasibility test. The intervention completion rate of each group will be assessed. Usability of the e-SMARPP website will be assessed using the Web Usability Scale (WUS) [49]. The WUS consist of 21 items measured on a 5-point scale and seven subscales: ease of use, ease in understanding structure, ease in reading, response speed, favorable, helpfulness and credibility. The subscale average score will be calculated, higher score indicates higher website usability. Usability of e-SMARPP contents will be assessed using original questionnaires. Example of questions include, “What is the most useful/unuseful content?”, “Are videos easy to use? (with options ranging from very easy to very difficult)” and “How long does it take to complete one exercise?”. Perceived program satisfaction will be assessed using the Client Satisfaction Questionnaire 8-item version (CSQ-8) [50]. CSQ-8 consists of eight items measured on a 4-point scale. A higher score indicates a higher satisfaction with service use.

#### Participant characteristics

At the baseline assessment, sociodemographic information will be gathered including age, sex, marital status, cohabitation status, educational history, employment status and Internet use (use days per week, hours per day and main devices to access).

Information about history of drug use will be also asked. The primary problematic drug will be asked. Drug use in the past 28 days will be collected using the self-monitoring calendar based on the TLFB method. In addition, we assess first-abused drug, onset age of any drug abuse and the primary drug abuse, polydrug abuse (yes/no), abstinence duration calculated from the day when they last used a drug, experience of past arrest (yes/no), past experience in a correctional facilities (yes/no), and self-reported psychiatric comorbidity with an option to select a diagnosis based on the International Classification of Diseases-10. Similarly, we evaluate history of treatment in several ways: duration of psychiatry outpatient ward, number of psychiatry hospitalization, specialized behavioral treatment for drug problems in the past (yes/no), and self-help group use (yes/no).

In order to assess severity of drug use problems, we use the Japanese version of the Drug Abuse Screening Test (DAST-20), which consists of 20 binary items [51, 52]. All items will ask participants’ drug use condition in the past year. Total score ranges from 0 to 20 and a high score represents a severe condition. The cutoff score for drug use disorders is suggested as 5/6 with maximum sensitivity and specificity [53–55], although an optimal cutoff score has not confirmed in different populations and culture. It is also suggested that a score of 16 or greater be considered to indicate a very severe dependence condition [56]. Furthermore, the Kessler-6 scale consisting of six items measured on a 5-point scale will be used to assess psychological distress [57, 58]. A total score ranging from 0 to 24 and a high score indicates severe distress. The optimal cut-off point is considered 4/5 for a mood and anxiety disorder [59].

### Sample size

Sample size is assumed for two primary outcome variables (relapse risk and the longest duration of abstinence) to detect a medium effect size of *d* = 0.4 based on previous studies for drug users. As for the first primary outcome (relapse risk), the effect size between pre and post intervention was *d* = 0.39 in a study conducting a relapse prevention program in Japan [60]. On another primary outcome (the longest duration of abstinence), the effect size between the intervention group and control group after the intervention was reported as *d* = 0.45 in a study conducting for computer-assisted cognitive behavioral therapy [19]. We estimated a sample size of 100 per group (total 200), assuming α = 0.05 and a power (1 - β) = 0.08. Attrition rate and non-completion rate was reported as relatively high (about 10–45 %) in previous studies of computer-assisted and web-based intervention for drug users [11, 19, 61]. However, we did not include additional samples because we expect a low attrition rate because all the participants will be outpatients motivated to seek treatment and we will send email reminders to follow up.

### Statistical analysis

#### Primary analysis

The primary analysis for the SRRS score will be on an intention-to-treat basis, using mixed-effect models. We will use all obtained data at the 2, 5 and 8 months assessment without imputation, assuming the missing mechanism will be at random given observed data within the groups. We will include the following variables as fixed effects: the group, time, the baseline scores and the interaction of group and time. Time will be coded as months after the baseline assessment, giving values of 0, 2, 5 and 8. We will also include random effects of participants for intercept and time. The effect of the intervention will be assessed by a test of hypothesis that a time and group interaction equals 0. To help interpretation in terms of effect size, Cohen’s *d* between groups and 95 % confidence intervals will be calculated at each assessment point; the values of 0.2, 0.5 and 0.8 are considered as small, medium and large effect, respectively [62]. The longest consecutive abstinent days from the primary abused drug during intervention will be compared using *t*-test. Also, we will calculate a Cohen’s *d* for the longest consecutive abstinent days.

When there are significant differences in both of the two primary outcomes between groups with a level of 5 % in the two-sided test, the intervention will be considered effective. Analyses will be conducted using SPSS Statistics Ver. 22.

#### Sensitivity analysis

In order to assess the sensitivity of the results due to the assumption of a missing mechanism, we will conduct complete case analysis using the inverse probability weighted generalized estimating equation (IPW-GEE), assuming the missing data will be missing at random given observed outcome and time-dependent covariates. IP weights at each assessment will be estimated by pooled logistic models for the probability of not dropping-out from the follow-up, conditional on the measured risk factors and groups; weights are the reciprocal of the estimated probability. Additionally, we will use IPW-GEE to assess effect of the intervention when the definition of abstinence is changed: (1) complete abstinence from all substances including the non-primary abused drug and alcohol and (2) the longest duration of consecutive abstinence in the past 28 days instead of the intervention period (56 days).

#### Subgroup analysis

The effect of the intervention will be assessed by subgroups because the effect may vary depending on specific population. The participants will be divided by the primary abused drug (i.e., methamphetamine, NPS and prescription drugs), sex, severity of drug addiction (DAST-20 score: 0–5, 6–15, 16–20), the duration of abstinence at the baseline assessment (<1 month, 1–6 months, 7–12 months) and whether receiving face-to-face behavioral therapy or not.

#### Cost effectiveness analysis

Health economic evaluation will be undertaken using data generated within the present trial, to provide information on the ‘value’ of allocating resources to the e-SMARPP (plus usual care) strategy over self-monitoring (plus usual care). The research question regarding the economic evaluation is, ‘Is e-SMARPP potentially a cost-effective means of helping abuse drug users to increase abstinent days? Specifically, we will employ a cost-effectiveness analysis (CEA) framework, and the results of analysis will be summarized as an incremental cost-effectiveness ratio (ICER). A within-trial analysis will evaluate cost-effectiveness from a patient and institutional perspective. Sensitivity analysis will be undertaken for parameter uncertainty of benefits measures and assumptions to calculate average costs. Subgroup analysis will be of great interest to make policy recommendations for a more targeted approach. Subgroups categorized by variables set in the main trail analysis, e.g., types of abuse drug primarily used, will be adopted for this subgroup analysis in an economic evaluation.

### Data monitoring

The research members who have an e-SMARPP account (AT, YM, NK and TM) will monitor the data. The first author (AT) will manage participants’ progress and completion of the intervention and the follow-up assessments. The members will share information about recruitment progress and data collection every month.

Information of adverse events including hospitalization, arrest and death will be collected from the participants’ primary doctor during the intervention. Additionally, participants will be asked about subjective harmful effect (i.e., craving, mental distress) when they use e-SMARPP at the follow-up assessment (T2).

### Ethics and dissemination

#### Research ethics and approval

The Ethics Committee of the Faculty of Medicine and Graduate School of Medicine of the University of Tokyo and the Ethics Committee of each recruiting hospital and clinic (National Center of Neurology and psychiatry, Saitama Psychiatric Medical Center, Kanagawa Psychiatric Center, Okayama Psychiatric Medical Center, Tokyo Metropolitan Matsuzawa Hospital and APARI clinic) approved this study. Before the baseline survey, candidates will be fully informed that their participation is totally voluntary and can withdraw the consent if they want and they can send a withdrawal e-mail to researchers and also tell their intention to withdraw to their primary doctor. Even if they withdraw the consent, they will not receive any disadvantage. In addition, they will be informed that the findings of this study will be disseminated without participants’ personal infromation via publication and website. Face-to-face informed consent will be conducted (AT) and signed consent forms will be obtained from all participants. The participants will be told that the web-based program does not provide emergency support verbally and on the website and will be encouraged to use proper medical services or talk to their primary doctor in case of an emergency. If a researcher becomes aware of an emergency condition (e.g., imminent suicide intention, violence) through e-SMARPP, the researcher will consult with the participant’s primary doctor. All data collected in this study is securely stored without the participants’ personal information (name, address, etc.). Access to the data is encrypted and limited to research staff named on the ethics protocol.

This study protocol was registered with the University Hospital Medical Information Network clinical trial registry (UMIN-CTR), number UMIN000016075. If there are important modifications of the protocol, we will obtain approval for the modifications from the Ethics Committee of the Faculty of Medicine and Graduate School of Medicine of the University of Tokyo and will revise the protocol on the UMIN-CTR website.

#### Dissemination of research findings

The study findings will be disseminated via publications in peer-reviewed international journals, the e-SMARPP website and a research report submitted to the Pfizer Health Research Foundation. We will also present the findings at relevant research conferences, local academic symposiums and seminars.

## Discussion

There are strengths to this study. First, to the best of our knowledge, this study is the first clinical trial using a web-based program for drug users in Japan. The randomized design and 8-month follow-up will allow for conclusive results. Second, the e-SMARPP contents were developed based on an existing evidence-based face-to-face program and usability has been confirmed. If successful, e-SMARPP will be promising approach to addressing problems of treatment implementation and will be useful for assisting drug user recovery without lower costs and ease of use. Finally, the study findings will be also useful for Asian countries where there are many amphetamine-type stimulants users and strong drug-use stigma. This circumstance in Japan reflects a different situation for drug abuse and policy from that of Western countries where many web-/mobile-based programs have been developed for that context.

There are possible limitations to this study. All data from participants will be self-reported and affected by situation and perception of participants. Regarding the program, the relapse prevention sessions of e-SMARPP requires the involvement of human resources, and web-therapists who give feedback comments to users’ homework. This raises concerns about scalability. More web-therapists and automated functions using algorithms to support a personalized program will be required when we disseminate e-SMARPP more widely. Additionally, it is important to consider whether e-SMARPP is useful and safe when recruiting drug users who do not receive other treatment and support. For now, we think e-SMARPP is an adjunct or a partial replacement for standard treatment. It is important to consider the possibility of implementation of e-SMARPP with collaboration from primary care and providers of mental health services. The Internet penetration rate is more than 90 % among people age 13 to 59 in Japan and it has been increasing year by year, but the rate is low among people with a low household income [63]. Individual Internet literacy and computer skills may affect not only usability and completion of the intervention, but also improvement of drug use problems in this study. Further revision may be required to provide a more use-friendly program.

### Availability of data and materials

A person who is interested in this study will communicate trial results via publications. The data collected in this study can be obtained from the first author upon request.

## Abbreviations

CSQ-8: client satisfaction questionnaire 8-item version
DAST-20: drug abuse screening test
IPW-GEE: inverse probability weighted generalized estimating equation
NPS: new psychoactive substances
QOL: quality of life
RCT: randomized controlled trial
SMARPP: Serigaya Methamphetamine Relapse Prevention Program
SOC: sense of coherence
SOCRATES-8D: stage of change readiness and treatment scale-8 version for drug use
SRRS: stimulant relapse prevention scale
SSDD: self-efficacy scale for drug dependence
TLFB: timeline followback
UMIN-CTR: University Hospital Medical Information Network clinical trial registry
